# Peripartum cardiomyopathy: a review of prevalence and treatment trends from an African perspective

**DOI:** 10.3389/fcvm.2025.1568493

**Published:** 2025-04-28

**Authors:** Kedir Negesso Tukeni, Elsah Tegene Asefa, Tamirat Godebo Woyimo, Esayas Kebede Gudina, Heidi Estner, Nikolaus Alexander Haas

**Affiliations:** ^1^ Medical Faculty, Ludwig Maximilians University, Munich, Germany; ^2^ Department of Internal Medicine, Jimma University, Jimma, Ethiopia; ^3^ Department of Cardiology, Ludwig Maximilians University, Munich, Germany; ^4^ Department of Pediatric Cardiology and Pediatric Intensive Care, Ludwig Maximilians University, Munich, Germany

**Keywords:** peripartum cardiomyopathy, diagnosis, Africa, review article, treatment patterns, treatment outcome, prevalence

## Abstract

Peripartum cardiomyopathy (PPCM) is a type of dilated cardiomyopathy that develops in women without a history of heart disease during the last trimester of pregnancy or within 6 months postpartum. It is one of the primary causes of heart failure during pregnancy, which increases peripartum morbidity and mortality. PPCM can cause significant left ventricular dysfunction, progressive heart failure, and refractory cardiogenic shock, resulting in increased maternal morbidity and mortality. Dyspnea, exhaustion, and lower extremity edema are common symptoms and are often misdiagnosed as normal postpartum changes, demanding careful assessment with echocardiography. Furthermore, diagnosis and treatment are often delayed due to insufficient awareness among healthcare providers, with varying definitions of the disease across countries. Its underlying causes remain unclear, although recent studies point to a potential prolactin-oxidative stress mechanism that might lead to potential future treatments. Clinical care follows basic heart failure management guidelines while taking medication teratogenicity into account. The prognosis varies geographically based on the level and pattern of treatment, with a considerable number of patients displaying partial recovery. The prevalence and treatment patterns of these patients in Africa, including the benefits and safety profiles of bromocriptine, are reviewed here, to identify directions in its prospective use in different forms of cardiomyopathies based on the available literature.

## 1 Introduction

Peripartum cardiomyopathy (PPCM) is a type of heart failure (HF) characterized by a significant decrease in left ventricular ejection fraction (LVEF) of <45%, occurring near the end of pregnancy or in the subsequent months after childbirth. Initially defined as occurring from the last month of pregnancy to 5 months postpartum, the understanding of PPCM has expanded to include cases that arise outside this original timeframe, rendering the definition time-independent (1, 2). Symptoms typically present within the first month postpartum and include dyspnea, lower extremity edema, and fatigue, which are often mistaken for normal postpartum changes (3–5). Diagnosis can be aided by echocardiography (2). PPCM can lead to severe impairment of left ventricular (LV) function, refractory cardiogenic shock, or advanced heart failure (HF) resulting in increased maternal morbidity and mortality, although the clear underlying pathogenesis is not straightforward forward, hence the area of active research (5–10). The disease is often diagnosed and treated late due to insufficient awareness among healthcare providers, with varying definitions across countries. While its underlying causes remain unclear, recent studies point to a potential prolactin-oxidative stress mechanism that might inform potential future treatments. Clinical management principles align with standard heart failure care while considering drug teratogenicity. Prognosis differs geographically, with approximately one-third of patients regaining normal cardiac function, although another third shows partial recovery (11–13).

In this review, we discuss the prevalence and treatment trends of patients with peripartum cardiomyopathy in Africa, including the benefits and safety profiles of bromocriptine, to highlight directions in its potential use in other types of cardiomyopathies. We will also focus on treatment outcomes among patients treated with bromocriptine on top of standard heart failure treatment.

## 2 Peripartum cardiomyopathy: epidemiology and disease prevalence in Africa

**Figure 1:**
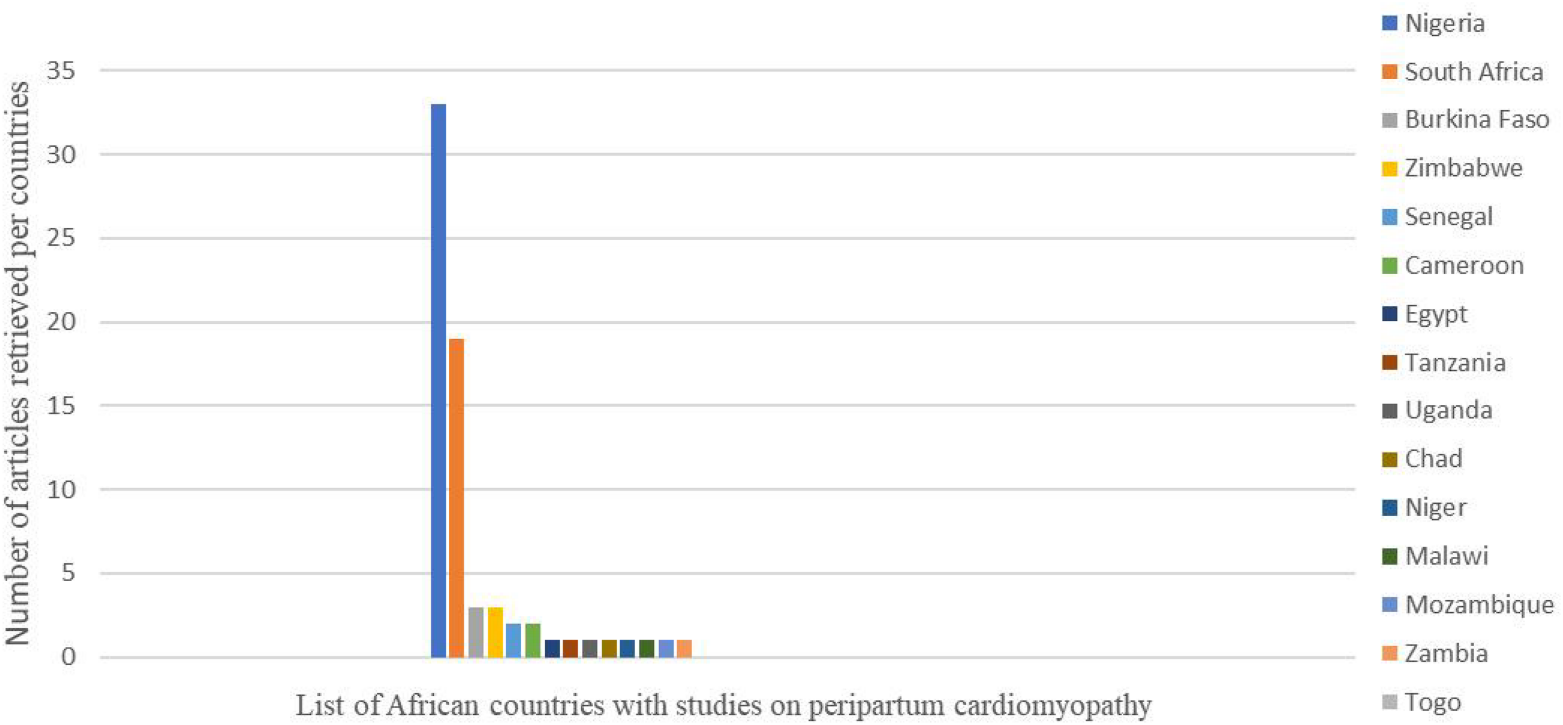
Peripartum cardiomyopathy in African countries: articles retrieved as per countries.

The epidemiology of peripartum cardiomyopathy might be underreported as the symptoms are often mistaken for normal postpartum changes leading to delayed diagnosis (3–5), necessitating the need for vigilant clinical evaluation with investigations including biomarkers and imaging with echocardiography. PPCM is the third cause of heart failure in pregnancy, only followed by hypertension and rheumatic heart disease (RHD) as demonstrated in a Nigerian study (5). Another Ethiopian study also showed that PPCM together with dilated cardiomyopathy (DCM) was also one of the leading causes of hospital admission in the setup (13). An in-hospital prevalence of PPCM was found to be 2.3% in another study, in which the average age of affected women was approximately 27 years. The patients had an average left ventricular end-diastolic diameter (LVEDD) of 58.9 mm, with an average left ventricular ejection fraction (LVEF) of 33.19% (14) (Figure 1).

**Figure 2:**
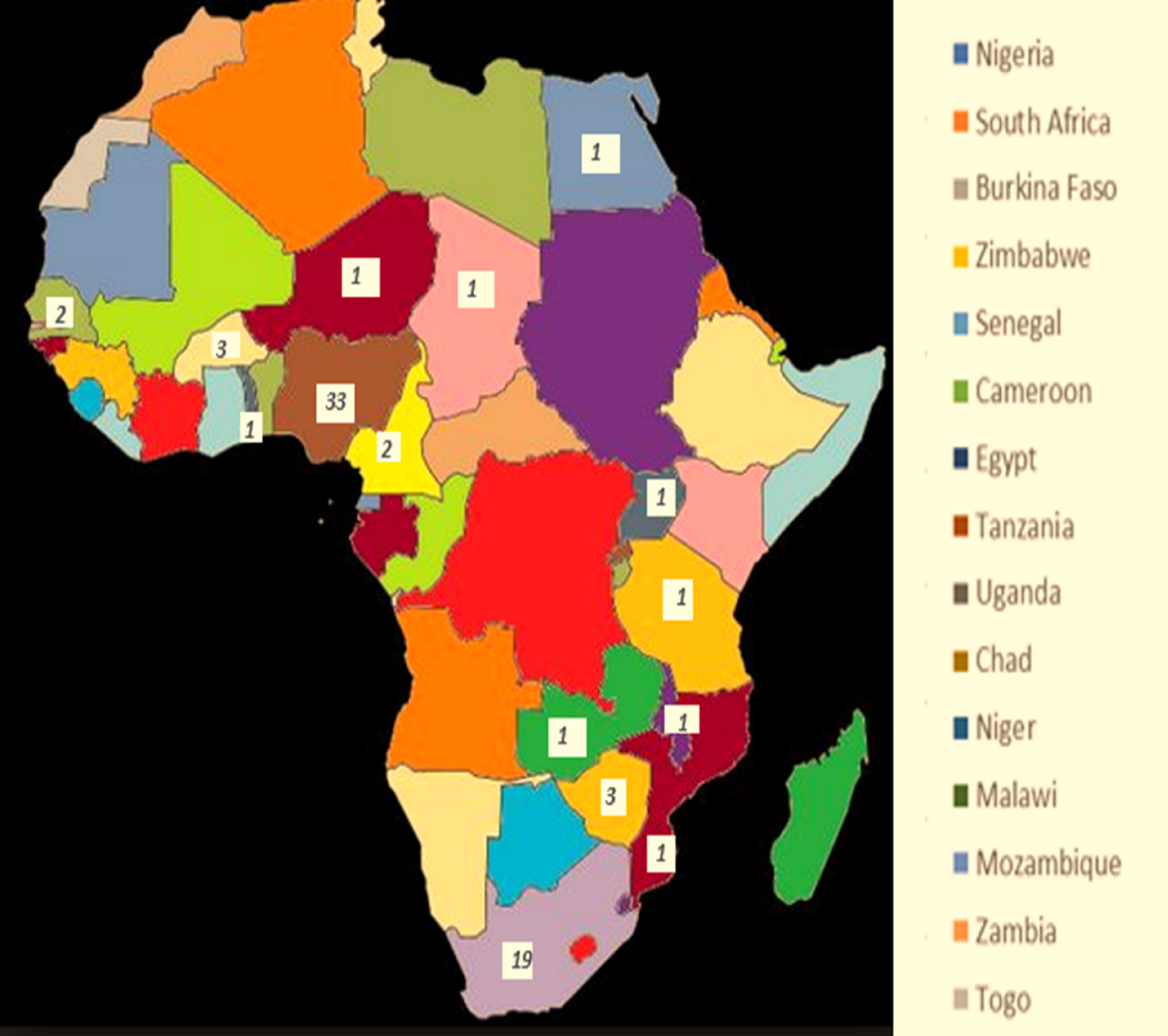
Peripartum cardiomyopathy: distribution of articles as per African countries.

Some studies report the prevalence of PPCM as high as 60% among women with heart failure, which led to approximately 34% of all peripartum deaths in the setting, while others report 1 in 1,000 births as was seen in the South African population. The incidence and disease burden varied substantially within the same region, as was seen in different geographical zones of Nigeria, reporting as high as 1 per 96 live births of PPCM (15, 16). According to an Egyptian study, PPCM is one of the primary causes of heart failure among hospitalized patients, with a prevalence of 6%, and was associated with a poor outcome (17, 18). Although the type and demographics of the study vary, there are significant burden of PPCM among mothers of childbearing age necessitating further study on disease pathogenesis and potential treatment alternatives in this resource-limited setting (Figure 2 and Table 1).

**Table 1:** Prevalence of peripartum cardiomyopathy in African studies.

Author	Year	Country	Number of cases	Prevalence of PPCM	Title of the article
Fundikira, et al.	2024	Tanzania	402	11.2%	Characterization of non-ischemic dilated cardiomyopathy in a native Tanzanian cohort: MOYO study
Nabbaale, et al.	2020	Uganda	236	17.4%	Burden, predictors and short-term outcomes of peripartum cardiomyopathy in a black African cohort
Kamdem, et al.	2023	Cameroon	2,102	1.3% (hospital prevalence)	Epidemiological features and mortality risk factors of peripartum cardiomyopathy in a group of sub-Saharan African population
Aw, et al.	2017	Senegal	326	5.8% (hospital prevalence)	Prevalence and characteristics of dysfunction of right ventricle in peripartum cardiomyopathy
Manga, et al.	2021	Senegal	32	2.3% (hospital prevalence)	Peri-partum cardiomyopathy: epidemiological, clinical aspects and risk factors in semi-urban areas in Senegal
Adib and Shahlazadeh	2015	Nigeria	8,100	1:1,000 (South Africa)	Prevalence of peripartum cardiomyopathy in pregnant women
				1:100 (Nigeria)	

## 3 Peripartum cardiomyopathy: etiology and potential risk factors

The risk factors for the development of PPCM and its outcomes are poorly understood. However, factors like multiparous and twin pregnancies were associated with the development of PPCM as was seen in the study that showed 43.7% and 28.1% of cases, respectively (14). A retrospective study that also looked at probable familial disease in individuals with dilated cardiomyopathy (DCM) and PPCM to determine the risk factors and predictors of survival outcome found 18 index patients with PPCM who had at least one first-degree cousin with DCM. Four of these families displayed autosomal dominant inheritance, while three showed autosomal recessive inheritance, indicating PPCM's position on the familial DCM spectrum (19–21). There is also the potential relationship between the development of PPCM and ABO-Rhesus, as was discovered from a study that reviewed a record of 90 consecutive patients, of whom the majority (55.1%) belonged to blood group B. It showed that there is a potential relationship of PPCM with blood group B, as they were more likely to develop PPCM as compared with others (22).

A Nigerian case–control study that included 39 patients with PPCM and 50 controls found that patients with PPCM had a significantly lower mean blood selenium level (61.7 ± 14.9 μg/L) than controls (118.4 ± 45.6 μg/L), indicating that selenium deficiency could also be a risk factor for the development of PPCM (7, 9).

Studies also suggest that humoral immunity could be a potential risk factor for the development of PPCM. The study that evaluated immunoglobulins against cardiac myosin in patients with PPCM from various global regions, including Africa, and compared them with healthy mothers and patients with idiopathic DCM as a potential etiologic factor, demonstrated that all PPCM groups had similar Ig profiles, which were significantly higher in PPCM patients compared with patients with idiopathic DCM. Furthermore, IgG3-positive individuals had a higher NYHA class at initial presentation, which may have predictive relevance in clinical PPCM (23). An increment in heart rate than usual also tends to increase the potential development of PPCM, as demonstrated in a case–control study of 54 PPCM and 77 controls that found a one-beat increase in heart rate increased the risk of PPCM by 6.4% and non-specific ST–T-wave changes also increased the odds of PPCM by 12.06-fold as compared with controls (23). Others including maternal age, multiparity, multiple gestations, obesity, chronic hypertension, African heritage, continuous use of tocolytics, singleton pregnancy, and gestational hypertension are related to an increased risk of developing PPCM as compared with their counterparts (10, 24, 25).

## 4 Peripartum cardiomyopathy: diagnostic workups in an African setting

PPCM is a diagnosis of exclusion considered when a pregnant woman presents with an LVEF of <45% during the peripartum period, provided there is no structural heart disease. Although (LV dilation is often observed, it should not solely be used for diagnosis as some enlargement can be a normal adaptation to pregnancy (1, 2). Other potential diagnoses that need to be ruled out include pre-existing structural heart conditions, pulmonary embolism, preeclampsia-related pulmonary edema, spontaneous coronary artery dissection, myocarditis, Takotsubo syndrome, myocardial infarction, aortic dissection with acute regurgitation, alcohol abuse, and effects of chemotherapeutic agents (2). Therefore, comprehensive clinical evaluation supported with imaging that includes chest x-rays, ECG, and echocardiography provides basic diagnostic investigations in the diagnosis of PPCM. However, ECG can present non-specific abnormalities, and a normal ECG does not eliminate the possibility of PPCM. A case–control study of 54 PPCM patients and 77 controls recruited consecutively found that a one-beat increase in heart rate increased the risk of PPCM by 6.4%, while the presence of ST–T-wave changes increased the odds of PPCM 12.06-fold compared with controls. A risk score of ≥2 [score 1 for each of the three ECG disturbances (tachycardia, ST–T-wave abnormalities, and QRS duration)] had a sensitivity of 85.2%, specificity of 64.9%, negative predictive value of 86.2%, and area under the curve of 83.8% for potentially predicting PPCM, directing the potential use of the risk score to help diagnose PPCM with significant accuracy, before confirmatory investigations in postpartum women (26). Another study, which looked at the prevalence of major and minor ECG abnormalities in PPCM patients at the time of diagnosis and whether there were ECG correlates of persistent left ventricular dysfunction and/or clinical stability at 6 months, included 78 patients with PPCM who had 12-lead ECGs performed at the time of diagnosis and 44 cases (56%) at 6 months. While ECG findings are not definitive for PPCM, they can effectively help exclude conditions such as ST-segment elevation myocardial infarction (1, 2).

Elevated levels of serum brain natriuretic peptide and its N-terminal portion are characteristic of peripartum cardiomyopathy (PPCM), while these levels remain normal during pregnancy or are only slightly increased in preeclampsia, and hence be used as one of the diagnostic criteria (2).

PPCM diagnosis primarily employs echocardiography, which evaluates the severity of left ventricular dysfunction; assesses right ventricular involvement, heart chamber sizes, and functional mitral or tricuspid regurgitation; and excludes left ventricular apical thrombosis in cases of significant left ventricular ejection fraction reduction (1, 27). Cardiac magnetic resonance is a viable option when echocardiographic windows are inadequate; however, the use of gadolinium should be avoided during pregnancy and only considered after childbirth (27).

Imaging modalities that use ionizing radiations should be avoided during pregnancy, unless necessary. Even if the hazards to the fetus are lessened during the third trimester, when PPCM usually occurs, ionizing radiation doses should be kept as low as reasonably achievable (27). Chest x-rays can be useful in determining the degree of pulmonary congestion, but lung ultrasonography should always be preferred when possible. Computed tomography may be required to rule out particular differential diagnoses, such as pulmonary embolism and aortic dissection (28). However, critical clinical evaluation with echocardiography and other investigations including chest x-rays, ECG, and biomarkers are widely used as more modern investigations are limited in a wider area in the continent.

Right ventricular involvement in the disease process is commonly reported, necessitating proper assessment of right ventricular function as it would be associated with poor patient outcomes as compared with their counterparts. This was demonstrated in a study of 19 PPCM patients that examined the prevalence of RV dysfunction. Every patient in the study had left ventricular dysfunction, with an average ejection fraction of 23.01%. Notably, 57.9% of the patients had RV systolic dysfunction, and there were significant abnormalities in various RV function metrics, including tricuspid annular plane systolic excursion (TAPSE) and fractional area change. As a result, there is a need for a complete investigation of RV parameters for improved identification and prognosis of patients with PPCM (29, 30). The RV involvement is even worse in some types of underlying risks, as was demonstrated in PPCM patients with selenium deficiency, where the RV insufficiency appears to be related to decreased late diastolic RV relaxation (31, 32). Furthermore, patients with RVSD had higher serum creatinine, and a large majority of the deceased patients (83.3%) had RVSD, showing the significance of RV dysfunction in patients with peripartum cardiomyopathy (29, 30, 33).

### 4.1 Peripartum cardiomyopathy: patient profiles

Patients with PPCM have divergent sociodemographics and are younger in most of the studies, with significantly dilated left ventricular internal dimensions. A prospective observational Zambian study that included 45 patients showed a mean age of 32.9 years, an average parity of 3.4 (SD:2.2), and twin pregnancies in 9% of cases. The baseline median LVEF was 36%, and the median left ventricular end-diastolic volume was 150 ml (IQR: 58-229). Seventy-nine of them had NYHA functional class IV symptoms, while 44% had associated gestational hypertension in which 22% had a history of preeclampsia (34). A prospective cohort study that evaluated 43 patients diagnosed with PPCM within 6 months, also demonstrated a mean age of 27.9 years and 34.9% being first-time mothers. The baseline LVEDD was 56.8 mm while LVEF was 29.7% (35).

In the cohorts of Germany that included German and South African patients, the South African patients displayed more profibrotic biomarker profiles that led to less favorable outcomes despite higher baseline LVEF at diagnosis (the baseline LVEF was 24 ± 8%, as compared with SA-PPCM patients with a baseline LVEF of 30 ± 9%) (36, 37).

A group of 54 patients with PPCM were evaluated for 1-year survival and left ventricular reverse remodeling (LVRR) at a 12-month follow-up. The LVRR was defined as an absolute increase in LVEF by ≥10.0% and a decrease in LVEDD indexed to body surface area ≤33.0 mm/m^2^, while recovered LV systolic function was defined as LVEF ≥55%. The study indicated that nearly half (47.1%) met the criteria for LVRR, with five (29.4%) recovering LV systolic function (LVEF ≥ 55%) (38). The *post hoc* analysis of the PEACE registry analysis found 90 (47.1%) had hyponatremia at presentation, with a mean serum sodium concentration of 126.7 ± 22.3 mmol/L, which had a threefold increase in all-cause mortality (15, 39) among the study participants.

Successive pregnancies (SSPs) of patients with PPCM have a high risk of heart failure relapse as was demonstrated in the study that included 34 PPCM patients with an SSP and found that the pregnancy ended prematurely in four patients. The overall recurrence rate (LVEF < 50% or death after at least 6-month follow-up) was 56%. Relapse of PPCM following SSP was not linked to variations in parity, twin pregnancy, gestational hypertension, or smoking. Before attending SSP, 47% of patients had persistently low LVEF (<50%), while 53% had full recovery (LVEF ≥ 50%) (40, 41).

In the ESC EURObservational Research Programme that enrolled 739 patients from 49 countries, including Africa, the average age was nearly similar, 31 ± 6 years. Their mean LVEF was 31% ± 10%, while 67% of the patients studied had an LVEF ≤ 35%. The majority of them were very symptomatic (functional class III/IV) at presentation, which happened during the first month of delivery in approximately 44% (42, 43). In a prospective study that included 49 patients newly diagnosed with PPCM, the average age at diagnosis was 28.1 years, with one-third being primigravida mothers. Approximately 35% experienced pregnancy-induced hypertension (44). A case series from Moroccan University Hospital involving five patients with PPCM also demonstrated that the condition predominantly affects younger women, with an average age of 32.6 years (45). Four of them had previously experienced normal multiple pregnancies without PPCM, except for one who suffered a recurrence after her fifth pregnancy. Upon admission, all exhibited significantly reduced LVEF, with varied presentations including acute heart failure or recurrent thromboembolic events with the onset being immediate postdelivery admissions (45).

## 5 Peripartum cardiomyopathy: treatment patterns and patient outcomes

### 5.1 Conventional treatment patterns in Africa

Treatment of heart failure due to PPCM follows standard guidelines that include but are not limited to beta-blockers and angiotensin-converting enzyme inhibitors with diuretics based on the fluid status of the patients (1, 27, 28). These and recent advances in the knowledge and treatment of peripartum cardiomyopathy including advanced therapy have resulted in much better outcomes for affected women (28, 46). However, these treatments are very limited in the continent, leading to suboptimal treatment of patients and hence relatively unfavorable outcomes. Furthermore, guideline-directed medical therapy (GDMT) itself is suboptimally utilized due mainly to availability, affordability, and limited expertise. This was seen from an Egyptian study that showed the patterns of guideline-directed medical therapy including ACEI/ARBs (which was used in 75%), BB (used in 54%), MRA (used in 91%), and digoxin (used in 65%), although it included diverging patients with heart failure (17, 18).

The GDMT utilization is even lower, at 40%, among patients treated for PPCM, in which BB was used at 40%, ACEi/ARBs at 42%, MRA at 22%, and diuretics at 40%, although later optimized to BB to 92%, ACEi/ARBs to 89%, and MRA to 74%, within 6 months. Eighty-four percent of them were seen at 6 months, of whom 3% of them remained symptomatic (NYHA class IV) (34). In another prospective cohort study that evaluated 43 patients diagnosed with PPCM within 6 months, ACE inhibitors/ARBs and mineralocorticoid receptor antagonists were used in a significant percentage, while fewer used beta-blockers (35). In another study, ACEi (or ARBs), beta-blockers, MRAs, diuretics, and digoxin were used in 34.8%, 23.4%, 91%, 86.1%, and 66.8%, respectively (47), leading to significant recovery of LVEF in 82.1% at 6 months, which was documented in those treated optimally with diuretics (100%) and ACE inhibitors (93.8%) (14). In the cohorts from Germany that included German and South African patients, all patients received standard heart failure therapy although anticoagulation was used in only 7% of SA-PPCM patients. Furthermore, German patients used bromocriptine together with standard treatment which might led to better outcomes (36, 37).

As a result, many investigations are presently underway, to potentially block the projected disease development processes. The study, which aimed to evaluate the effectiveness and safety of any intervention (including bromocriptine) for the care of mothers with peripartum cardiomyopathy, ended up with an insufficient result, although there was promising evidence for bromocriptine treatment (48). Furthermore, individuals with peripartum cardiomyopathy and low ejection fraction (EF) are at a higher risk of ventricular arrhythmias and sudden cardiac death (SCD), which requires the timely use of implantable cardioverter-defibrillator (ICD) placement. Cardiogenic shock, although uncommon, necessitates immediate mechanical support. Although short-term assistance is available based on the patient's condition, the services are very limited in most of the African setup (Table 2).

**Table 2:** Summary of the number of articles retrieved from African countries showing mortality rates among patients with PPCM.

Author	Year	Country	Number of cases	Article title	Mortality rate	Bromocriptine use
Matemvu, et al.	2023	Malawi	5	Five cases of peripartum cardiomyopathy in Malawi	Five cases of peripartum cardiomyopathy: the details can be found in the body of this manuscript	The bromocriptine was used in two of them
Kamdem, et al.	2023	Cameroon	2,102	Epidemiological features and mortality risk factors of peripartum cardiomyopathy in a group of sub-Saharan African population	Mortality rate was 27.7%, with age <30 years associated with mortality	Not documented
Strasserking, et al.	2022	Zambia	45	Peripartum cardiomyopathy: characteristics and outcomes among women seen at a referral hospital in Lusaka, Zambia	7%	Noted documented
Munyandu, et al.	2017	Zimbabwe	43	Peripartum cardiomyopathy among cardiovascular patients referred for echocardiography at Parirenyatwa Teaching Hospital, Harare, Zimbabwe	11.6% (at 6 months)	Not reported
Yaméogo, et al.	2018	Burkina Faso	29	Maternal and fetal prognosis of subsequent pregnancy in black African women with peripartum cardiomyopathy	48.3%, SSP outcomes are still severe with maternal mortality remaining high	Not reported
Yaméogo, et al.	2017	Burkina Faso	96	Bromocriptine in management of peripartum cardiomyopathy: a randomized study on 96 women in Burkina Faso	This study assessed the role of bromocriptine in the management of peripartum cardiomyopathy	Randomized 96 PPCM patients with bromocriptine treatment
Seghda, et al.	2020	Burkina Faso	66	Prognosis of peripartum cardiomyopathy in sub-Saharan Africa (Burkina Faso South-West PPCM register)	Mortality rate was 13.3%, with delayed diagnosis related to non-recovery	Not reported

### 5.2 Advanced therapy and future directions

Device therapy including implantable cardioverter defibrillators and cardiac transplantations is among the advanced therapies for advanced-type patients with PPCM, usually after careful stabilization to protect fetal health, with treatment tailored to the severity of cardiac dysfunction. Additionally, outcomes for posttransplantation of patients with PPCM appear to be poorer compared with other cardiac transplant recipients, highlighting the challenges in managing this condition and the need for specialized care and research into alternative effective therapies, of which some are ongoing (4, 14, 36, 39–41). Unfortunately, these services are lacking in African settings, necessitating further studies as alternative treatment options.

One of the potential advanced therapies that is currently an area of active research is the use of bromocriptine in this group of patients. The fact that one of the proposed mechanisms of the development of PPCM involves cardiac injury from prolactin breakdown products leads to bromocriptine as a potential and, of course, promising treatment alternative for the disease. Its ability to inhibit degradation and its possible anti-inflammatory properties make it a significant focus of ongoing studies in this area. For instance, one of the randomized trials evaluated the role of bromocriptine in the management of PPCM. The patients were assigned to standard heart failure therapy (Br−) and compared with another arm that was randomized for bromocriptine (Br+) on top of standard heart failure therapy plus bromocriptine provided as 2.5 mg twice daily for 4 weeks. It comprised 96 women with a mean LVEDD of 58.7 mm in Br+ and 57.6 mm in Br−, a mean LVEF of 37.2% in Br+ and 37.5% in Br−, and a mean TAPSE of 19.9 mm in Br+ and 18.9 mm in Br− at baseline. At 6 months, the cumulative death rate remained at 8 (16.6%) in Br+ and 14 (29.1%) in Br−. Furthermore, echocardiographic measurements showed that Br+ had greater ventricular function, with a mean LVEDD of 53.4 mm, an LVEF of 49.9%, and a mean TAPSE of 22.0 mm. These measures increased significantly in the subsequent follow-up at 12 months, reaching a mean LVEF of 53% (while it was 45.9% in the control arm), LVEF of 53.9% (40.9% in the control arm), and mean TAPSE of 22.7 mm (was 20.9 mm in control arm), demonstrating the overall benefit of bromocriptine in the treatment of PPCM patients (37). For instance, in the cohorts from Germany that included German and South African patients, in which their baseline parameters and conventional therapy were similar, those in G-PPCM demonstrated better outcomes as there was attainment of full recovery in approximately 52% with no report of mortality while in the SA-PPCM demonstrated full recovery in <32% with high mortality rate of 11%. The use of bromocriptine and anticoagulation therapy in G-PPCM patients might counteract fibrosis leading to better outcomes (36, 37). The potential effect of bromocriptine in better patient outcomes is also demonstrated by a study that focused successive pregnancies in patients with PPCM could have a high risk of heart failure relapse with increased mortality. In this study, the concomitant use of bromocriptine with standard medication for heart failure immediately after delivery demonstrated significantly superior LVEF at follow-up and a greater incidence of full recovery with no patient dying as compared with patients receiving standard therapy for heart failure alone (40, 41). However, the use of bromocriptine is limited as was demonstrated in in the ESC EURObservational Research Programme that enrolled 739 patients from 49 countries, including Africa. It showed only in 15% of patients, indicating the need for further focus on this potential treatment of peripartum cardiomyopathy (42, 43).

### 5.3 Patient outcomes in an African setting

As a result of limitations both to conventional and more advanced therapy, outcomes of patients with PPCM are poor. The study that followed patients for 6 months following the diagnosis and initiation of GDMT found a mortality rate of 7% among patients, of whom 3% remained symptomatic (NYHA class IV). GDMT was provided only for 40% of them (36). The mortality was even higher in other studies, with an overall mortality rate of 27%, among these specific groups of African patients (17, 18), highlighting the importance of raising awareness further and implementing effective management strategies to reduce morbidity and mortality associated with PPCM (46, 49). In a prospective cohort study that evaluated 43 patients diagnosed with PPCM within 6 months, mortality was 11.6% although some of the GDMT were used at nearly optimal level, underscoring the serious risks associated with PPCM (35). The mortality is more prevalent among impoverished rural African populations as compared with those in Western Europe and North America, reaching up to 24.2% at 6 months and 47.4%, as was demonstrated in studies from Nigeria, Burkina Faso, Zimbabwe, and South Africa, indicating a serious public health concern in the setting (50–52). Furthermore, a significant number of patients with the disease have poor outcomes, as evidenced by a study that found nearly a fifth of patients died during a 6-month follow-up, in which younger age, tachycardia, hypotension, and low LVEF were associated with a poorer prognosis (14).

This poor patient outcome in those of African origin was also evidenced by another study focusing on the prognosis of PPCM patients in the African American population, who had been followed for a minimum of 24 months. The study found that mortality was high (15.9%) while LV function recovery was also low (documented in only 35%), although patients had similar treatment backgrounds to similar patients of different origins (53). Twin pregnancy, heart rate >120 bpm, presence of atrial fibrillation, use of warfarin, and persistent higher NYHA functional class at last visit were predictors of increased mortality (43). Moreover, low systolic blood pressure and high resting heart rate were associated with poor patient outcomes, as this led to lower appetite to use ACEi and beta-blockers, resulting in increased mortality at 6 months (54). Similarly, increased left ventricular end-systolic dimension (LVESD), lower BMI, and lower total cholesterol at baseline were also found to be predictors of prognosis in another study (55). Furthermore, hyponatremia is not only common in those with PPCM but also has a threefold increase in all-cause mortality (15, 39). Others including SBP <90 mmHg at presentation were associated with a fourfold increase in all-cause mortality, while lower blood sodium levels, eGFR, and left ventricular ejection fraction were also linked to higher in-hospital mortality as demonstrated from the *post hoc* analysis of the PEACE registry analysis (15, 39).

Optimal treatment with conventional therapy improves patient outcomes despite the baseline level of LV function and the underlying potential etiology, as was demonstrated in the cohorts from Germany including German and South African patients. Although the baseline LVEF was lower in G-PPCM patients, their outcome was better (full recovery, 52%; mortality, 0%) as compared with SA-PPCM patients (full recovery, 32%; mortality, 11%). The study concluded that SA-PPCM patients displayed a more profibrotic biomarker profile, which was associated with a less favorable outcome despite better cardiac function at baseline, and the use of bromocriptine and anticoagulation therapy in G-PPCM patients might also counteract fibrosis leading to better outcomes (36, 37).

The study which evaluated 54 patients with PPCM for 1-year survival and left ventricular reverse remodeling (LVRR) at a 12-month follow-up found high mortality despite nearly half (47.1%) meeting the criteria for LVRR (38). Successive pregnancies (SSPs) of patients with PPCM have also a high risk of heart failure relapse with increased mortality, 12% in one study (40, 41). Furthermore, in a woman who experienced PPCM and subsequently became pregnant again, mortality was even, at 44.8%, with a negative correlation found between LVEF at admission and mortality rates. This study highlighted that subsequent pregnancy outcomes were severe, contributing to an increased risk of maternal death (41).

A case series from Moroccan University Hospital involving five patients with PPCM demonstrated significantly reduced left ventricular ejection fractions, with varied presentations including acute heart failure or recurrent thromboembolic events. Although the patients were treated with GDMT, which led to improvement in some patients, others suffered severe consequences, including fatalities. This emphasizes the importance of early diagnosis, collaborative care, effective treatment strategies, and ongoing monitoring to enhance survival rates among affected mothers (45) (Table 3).

**Table 3:** Summary of case series of patients with PPCM at University Hospital in Morocco.

Patient	Age	Parity	NYHA class	LVEF	LVEDD	Medication
1	35	2	IV	21	61	Diuretics, ACEi, and low-dose beta-blockers
2	28	3	III	17	61	Inotropes, then diuretics, ivabradine, beta-blockers, and MRA
3^a^	30	3	IV	20	58	Diuretics, MRA, ACEi, and low-dose beta-blockers
4	36	4	IV	22	59	Diuretics, ACEi, MRA and low-dose beta-blockers
5^a^	34	5	IV	20	60	Diuretics, ACEi, beta-blockers, MRA

^a^ Both of them died despite treatment.

In the ESC EURObservational Research Programme that enrolled 739 patients from 49 countries, including 29% from Africa, the total 6-month mortality rate was 6%, and the majority were due to heart failure (42%), with sudden death accounting for 30%. Patients with LVEF > 0.30 have a high recovery rate, while those with severe dysfunction (LVEF < 0.30) and significant ventricular dilation face lower odds of recovery and require innovative therapeutic strategies (42, 43).

In a prospective study that included 49 patients newly diagnosed with PPCM, approximately 16.3% died within 2 years, although nearly half of the patients (49%) fully regained left ventricular function. Younger age, higher functional heart failure class at presentation, and lower initial LVEF levels are associated with increased maternal mortality (44). Late diagnosis and surveillance were linked to poor recovery of left ventricular function in patients with peripartum cardiomyopathy and led to increased (as high as 13.3%) maternal mortality as well (56). In a prospective cohort study, the PEACE registry that compared 406 consecutive PPCM patients to 99 healthy pregnant women reported an 18.7% mortality rate alongside low rates of left ventricular reverse remodeling (24.1%) and functional recovery (22.6%). Although significant selenium deficiency was identified in 84.9% of patients, supplementation did not lead to improved all-cause mortality or left ventricular recovery outcomes, and in another study, no correlation was found between selenium levels and cardiac function or geometry at all (15, 39).

A case series from Moroccan University Hospital involving five patients with PPCM, who had significantly reduced LVEF, resulted in the death of two of them (45).

## 6 Conclusion

Peripartum cardiomyopathy is one of the leading causes of pregnancy-related maternal mortality in Africa. The management course and medications vary by region, with beta-blockers and angiotensin-converting enzyme inhibitors or angiotensin receptor blockers being the most commonly used, along with some uses of bromocriptine. Although its use is largely limited due to insufficient evidence, those who used bromocriptine experienced both better symptom improvement and LVEF recovery with no significant documented adverse effects. While a systematic review and meta-analysis are still required, the use of bromocriptine in this type of cardiac disease might improve patient outcomes with fewer adverse events than previously thought.

## References

[1] Sliwa K Hilfiker-Kleiner D Petrie MC Mebazaa A Pieske B Buchmann E Current state of knowledge on aetiology, diagnosis, management, and therapy of peripartum cardiomyopathy: a position statement from the heart failure association of the European Society of Cardiology working group on peripartum cardiomyopathy. Eur J Heart Fail*.* (2010) 12(8):767–78. 10.1093/eurjhf/hfq120 20675664

[2] Iannaccone G Graziani F Kacar P Tamborrino PP Lillo R Montanaro C Diagnosis and management of peripartum cardiomyopathy and recurrence risk. Int J Cardiol Congenit Heart Dis. (2024) 17:100530. 10.1016/j.ijcchd.2024.100530 39711771 PMC11657248

[3] Adib A Shahlazadeh H. Prevalence of peripartum cardiomyopathy in pregnant women. J Isfahan Med Sch. (2015) 33(353):1686–90.

[4] Roldán MC. Peripartum cardiomyopathy | cardiomiopatia periparto | miocardiopatía periparto. Insuficiencia Cardiaca. (2022) 17(2):55–60.

[5] Berliner D Li T Mariani S Hamdan R Hanke J König T Clinical characteristics and long-term outcomes in patients with peripartum cardiomyopathy (PPCM) receiving left ventricular assist devices (LVAD). Artif Organs. (2023) 47(2):417–24. 10.1111/aor.14406 36113950

[6] Saidu H Kabir A Ndiche N Yau JA Abdullahi U Mijinyawa MS. Prevalence and characteristics of peripartum cardiomyopathy among women with cadiac failure referred for echocardiography in a tertiary hospital in northern Nigeria. J Biosci Med (Irvine). (2018) 06(03):25–35. 10.4236/jbm.2018.63007

[7] Karaye KM Yahaya IA Lindmark K Henein MY. Serum selenium and ceruloplasmin in Nigerians with peripartum cardiomyopathy. Int J Mol Sci. (2015) 16(4):744–50. 10.3390/ijms16047644 PMC4425040 25853263

[8] Tobias SL Van der Westhuyzen J Davis RE Icke GC Atkinson PM. Alcohol intakes and deficiencies in thiamine and vitamin B6 in black patients with cardiac failure. S Afr Med J. (1989) 76(7):299–302. 2799573

[9] Cénac A Touré K Diarra MB Sidibé A Traoré A Coulibaly S Plasma selenium and peripartum cardiomyopathy in Bamako, Mali. Med Trop (Mars)*.* (2004) 64(2):151–4. 15460143

[10] Al Riyami N Al Khayari S Al Zadjali R Machado L Al Madhani A Al Lawati H. Incidence, risk factors, maternal and neonatal outcomes of peripartum cardiomyopathy (PPCM) in Oman. Glob Heart. 2023;18(1). 10.5334/gh.1198 37153846 PMC10162354

[11] Billson J Vollmer L. Peripartum cardiomyopathy: a review of the literature. Obstet Gynaecol Forum. (2014) 24(1):12–8.

[12] Karaye KM Henein MY. Peripartum cardiomyopathy: a review article. Int J Cardiol. (2013) 164(1):33–8. 10.1016/j.ijcard.2011.11.069 22192299

[13] Capriola M. Peripartum cardiomyopathy: a review. Int J Womens Health. (2012) 5(1):1–8. 10.2147/IJWH.S37137 23300351 PMC3536353

[14] Manga SJ Mohamed D Sy SL Te Indafa Q. Peri-partum cardiomyopathy: epidemiological, clinical aspects and risk factors in semi-urban areas in Senegal. OAlib. (2021) 08(11):1–8. 10.4236/oalib.1108050

[15] Karaye KM Ishaq NA Sa’idu H Balarabe SA Talle MA Isa MS Incidence, clinical characteristics, and risk factors of peripartum cardiomyopathy in Nigeria: results from the PEACE registry. ESC Heart Fail*.* (2020) 7(1):236–44. 10.1002/ehf2.12562 PMC7083508 31990449

[16] Karaye KM. Learning from the peripartum cardiomyopathy in Nigeria (PEACE) registry: a multisite, contemporary PPCM registry in Nigeria. In: Sliwa, K, editor. Peripartum Cardiomyopathy: From Pathophysiology to Management. London, San Diego, Cambridge (MA), Oxford: Elsevier (2021) p. 123–35. 10.1016/B978-0-12-817667-2.00011-6

[17] Reham Awad R Bassem I Osama L Rania H Hossam Abd-El A. Medium term prognosis of Egyptian patients hospitalised with acute decompansated heart failure. Eur J Heart Fail. (2016) 18(SUPPL. 1):450–5.

[18] Mandi DG Bamouni J Naïbé DT Yaméogo RA Kambiré Y Kologo KJ Epidemiology and long-term prognosis of atrial fibrillation in rural African patients. Egypt Heart J*.* (2019) 71(1):6. 10.1186/s43044-019-0005-3 31659514 PMC6821409

[19] Tibazarwa K Sliwa K Wonkam A Stevens J Boulle A Mayosi B. Familial aggregation of dilated cardiomyopathy in patients with peripartum cardiomyopathy. Circulation. (2012) 125(19):2307–15.

[20] Burkett EL Hershberger RE. Clinical and genetic issues in familial dilated cardiomyopathy. J Am Coll Cardiol. (2005) 45(7):814–8. 10.1016/j.jacc.2004.11.066 15808750

[21] Peters S Johnson R Birch S Zentner D Hershberger RE Fatkin D. Familial dilated cardiomyopathy. Heart Lung Circ. (2020) 29(4):535–41. 10.1016/j.hlc.2019.11.018 31974027

[22] Hayatu U Alabi IS Zagga MU Bello SO Yusuf SM Ibrahim A ABO-Rhesus blood group distribution among peripartum cardiomyopathy patients: a multi-center study in Sokoto, Nigeria. Int J Clin Cardiol*.* (2020) 7(3):182. 10.23937/2378-2951/1410182

[23] Warraich RS Sliwa K Damasceno A Mayosi BM Ntsekhe M Ojji D Impact of pregnancy-related heart failure on humoral immunity: clinical relevance of G3-subclass immunoglobulins in peripartum cardiomyopathy. Am Heart J*.* (2005) 150(2):263–9. 10.1016/j.ahj.2004.09.008 16086928

[24] Sharkey M Boydstun N. Peripartum cardiomyopathy. “Babies are breaking my heart!” In: Case Studies in Emergency Medicine: LEARNing Rounds: Learn, Evaluate, Adopt, Right now. Cham: Springer (2019) p. 123–8. 10.1007/978-3-030-22445-5_50

[25] Kane A Dia AA Diouf A Ba SA Bodian M Mbaye A Peripartum cardiomyopathy: a prospective echocardiographic study. Ann Cardiol Angeiol (Paris)*.* (2001) 50(6):305–11. 10.1016/S0003-3928(01)00037-3 12555620

[26] Karaye KM Lindmark K Henein MY. Electrocardiographic predictors of peripartum cardiomyopathy. Cardiovasc J Afr. (2016) 27(2):66–72. 10.5830/CVJA-2015-092 27213852 PMC4928165

[27] Regitz-Zagrosek V Roos-Hesselink JW Bauersachs J Blomström-Lundqvist C Cífková R De Bonis M 2018 ESC guidelines for the management of cardiovascular diseases during pregnancy. Eur Heart J*.* (2018) 39(34):3165–241. 10.1093/eurheartj/ehy340 30165544

[28] Davis MB Arany Z McNamara DM Goland S Elkayam U. Peripartum cardiomyopathy: jACC state-of-the-art review. J Am Coll Cardiol. (2020) 75(2):207–21. 10.1016/j.jacc.2019.11.014 31948651

[29] Aw F Ndiaye MB Sarr SA Bodian M Ngaide AA Mbaye A Prevalence and characteristics of dysfunction of right ventricle in peripartum cardiomyopathy. Res Rep Clin Cardiol*.* (2017) 8:61–6. 10.2147/RRCC.S141572

[30] Karaye KM. Right ventricular systolic function in peripartum and dilated cardiomyopathies. Eur J Echocardiogr. (2011) 12(5):345–50. 10.1093/ejechocard/jer024 21414954

[31] Karaye KM Lindmark K Henein M. OC10_08 prevalence and predictors of right ventricular diastolic dysfunction in peripartum cardiomyopathy patients. Glob Heart. (2016) 11(2):123–7. 10.1016/j.gheart.2016.03.046 28247237

[32] Karaye KM Lindmark K Henein MY. Prevalence and predictors of right ventricular diastolic dysfunction in peripartum cardiomyopathy. J Echocardiogr. (2017) 15(3):123–9. 10.1007/s12574-017-0333-9 28247237

[33] Karaye KM Lindmark K Henein M. Right ventricular systolic dysfunction and remodelling in Nigerians with peripartum cardiomyopathy: a longitudinal study. BMC Cardiovasc Disord. (2016) 16(1):1–8. 10.1186/s12872-016-0204-8 26821537 PMC4731908

[34] Strasserking FE Musho J Heimburger DC Mutale W Damp JA Mumba N Peripartum cardiomyopathy: characteristics and outcomes among women seen at a referral hospital in Lusaka, Zambia. Int J Cardiol Heart Vasc*.* (2022) 42:101104. 10.1016/j.ijcha.2022.101104 36046756 PMC9421395

[35] Gambahaya ET Hakim J Kao D Munyandu N Matenga J. Peripartum cardiomyopathy among cardiovascular patients referred for echocardiography at Parirenyatwa Teaching Hospital, Harare, Zimbabwe. Cardiovasc J Afr. (2017) 28(1):10–5. 10.5830/CVJA-2016-043 PMC5423423 28262909

[36] Azibani F Pfeffer TJ Ricke-Hoch M Kramer B Katus HA Sliwa K Outcome in German and South African peripartum cardiomyopathy cohorts associates with medical therapy and fibrosis markers. ESC Heart Fail*.* (2020) 7(2):512–22. 10.1002/ehf2.12553 32064780 PMC7160487

[37] Yaméogo NV Kagambèga LJ Seghda A Owona A Kaboré O. Bromocriptine in management of peripartum cardiomyopathy: a randomized study on 96 women in Burkina Faso. J Cardiol Clin Res. (2017) 5(2):1–7.

[38] Karaye K Lindmark K Henein M. One year survival in Nigerians with peripartum cardiomyopathy. Heart Views. (2016) 17(2):45–50. 10.4103/1995-705x.185114 PMC4966209 27512533

[39] Karaye KM Mohammed IY Sa’idu H Balarabe SA Talle MA Isa MS Prevalence of left ventricular dysfunction and relationship with serum selenium in apparently healthy pregnant women: results from the PEACE registry. Int Cardiovasc Forum J*.* (2020) 20:687. 10.17987/icfj.v20i0.687

[40] Hilfiker-Kleiner D Haghikia A Masuko D Nonhoff J Bauersachs J Sliwa K Outcome of subsequent pregnancies in patients with a history of peripartum cardiomyopathy. Eur J Heart Fail*.* (2017) 19(12):1723–8. 10.1002/ejhf.808 28345302

[41] Yaméogo NV Samadoulougou AK Kagambèga LJ Kologo KJ Tiemtoré-Kambou B Doulougou B Maternal and fetal prognosis of subsequent pregnancy in black African women with peripartum cardiomyopathy. BMC Cardiovasc Disord*.* (2018) 18(1):119. 10.1186/s12872-018-0856-7 29914408 PMC6006934

[42] Sliwa K Petrie MC van der Meer P Mebazaa A Hilfiker-Kleiner D Jackson AM Clinical presentation, management, and 6-month outcomes in women with peripartum cardiomyopathy: an ESC EORP registry. Eur Heart J*.* (2020) 41(39):3787–97. 10.1093/eurheartj/ehaa455 32840318 PMC7846090

[43] Ntusi NB Mayosi BM. Risk factors for disease development and predictors of outcome in peripartum cardiomyopathy. Eur Heart J. (2010) 31:2838–43.

[44] Gambahaya ET Hakim JG Kao DP Fana GT Matenga JA. Clinical characteristics and long term outcome of peripartum cardiomyopathy in a resource limited setting. Eur J Heart Fail. (2016) 18:654–9.

[45] Bouhaddoune Y Hbali A Aissaoui H Mrabet A Ismaili N El Ouafi N. Peripartum cardiomyopathy: alluring challenge-case series and review of literature. Pan Afr Med J. (2021) 40:1–10. 10.11604/pamj.2021.40.119.29168 PMC8627143 34887993

[46] Fett JD Markham DW. Discoveries in peripartum cardiomyopathy. Trends Cardiovasc Med. (2015) 25(5):401–6. 10.1016/j.tcm.2014.10.019 25557957

[47] Karaye KM Sa’idu H Balarabe SA Talle MA Isa MS Sani MU Clinical features and outcomes of peripartum cardiomyopathy in Nigeria. J Am Coll Cardiol*.* (2020) 76(20):2352–64. 10.1016/j.jacc.2020.09.540 33183509

[48] Carlin AJ Alfirevic Z Gyte GM. Interventions for treating peripartum cardiomyopathy to improve outcomes for women and babies. Cochrane Database Syst Rev. (2010) (9):CD008589. 10.1002/14651858.cd008589.pub2 20824881 PMC4170903

[49] Kwan GF Bukhman AK Miller AC Ngoma M Gishoma C Mukherjee JS A simplified echocardiographic strategy for heart failure diagnosis and management within an integrated noncommunicable disease clinic at district hospital level for sub-Saharan Africa. JACC Heart Fail*.* (2013) 1(3):230–6. 10.1016/j.jchf.2013.03.006 24621875

[50] Karaye K Habib A Sliwa K. Epidemiology of peripartum cardiomyopathy in Africa. Int Cardiovasc Forum J. (2019) 15:1–5. 10.17987/icfj.v15i0.545

[51] Sliwa K Mayosi BM. Recent advances in the epidemiology, pathogenesis and prognosis of acute heart failure and cardiomyopathy in Africa. Heart. (2013) 99(18):1317–22. 10.1136/heartjnl-2013-303592 23680887

[52] Karaye KM Sa’idu H Balarabe SA Talle MA Isa MS Sani MU Regional disparities in the clinical profiles of patients with peripartum cardiomyopathy in Nigeria: results from the peripartum cardiomyopathy in Nigeria registry. Eur Heart J*.* (2020) 41(Suppl_2):ehaa946.2068. 10.1093/ehjci/ehaa946.2068

[53] Modi KA Illum S Jariatul K Caldito G Reddy PC. Poor outcome of indigent patients with peripartum cardiomyopathy in the United States. Am J Obstet Gynecol. (2009) 201(2):171.e1–e5. 10.1016/j.ajog.2009.04.037 19564021

[54] Libhaber E Sliwa K Bachelier K Lamont K Böhm M. Low systolic blood pressure and high resting heart rate as predictors of outcome in patients with peripartum cardiomyopathy. Int J Cardiol. (2015) 190:376–82. 10.1016/j.ijcard.2015.04.081 25966297

[55] Blauwet LA Libhaber E Forster O Oelofse M Mertens L Vorster A Predictors of outcome in 176 South African patients with peripartum cardiomyopathy. Heart*.* (2013) 99(5):308–13. 10.1136/heartjnl-2012-302760 23118348

[56] Taryètba André AS Théodore B Jean EB Ouédraogo G Bazié F Kaboré B Prognosis of peripartum cardiomyopathy in sub-Saharan Africa (Burkina Faso south-west PPCM register). J Cardiol Cardiovasc Med*.* (2020) 5(2):1–8. 10.29328/journal.jccm.1001096

