# Supplementary material for: Peripartum cardiomyopathy: a review of prevalence and treatment trends from an African perspective
Source: Front Cardiovasc Med. 2025 Apr 28;12:1568493. doi: 10.3389/fcvm.2025.1568493 (PMC12066673; doi:10.3389/fcvm.2025.1568493)
Supplement: Supplementary file 1 [file Datasheet1.pdf]

Title: **Peripartum cardiomyopathy: A review of prevalence and treatment trends from an African perspective**

Table 1: Number of articles retrieved from the 54-African Countries

Search platform: **Scopus**

Search keywords: Peripartum cardiomyopathy (PPCM)+ (per Lists of African countries),

Article searched (Duration of publication): till November 17th, 2024

**S. No**	**List of African Countries**	**Number of articles retrieved**
1	Algeria	None
2	Tunisia	None
3	Libya	None
4	Egypt	One
5	South Africa	29(clear 19)
6	Tanzania	One
7	Ghana	None
8	Botswana	None
9	Namibia	None
10	Uganda	One
11	Ethiopia	None
12	Kenya	None
13	Eritrea	None
14	Rwanda	None
15	Sudan	None
16	South Sudan	None
17	Chad	One
18	DRC	None
19	Congo Brazzaville	None
20	Nigeria	45 (33=2 book section)
21	Niger	One
22	Mali	None
23	Malawi	One

24	Lesotho	None
25	Mozambique	Two
26	Cameroon	Two
27	Gambia	None
28	Zambia	One
29	Angola	None
30	Ivory Coast	None
31	Togo	One
32	Senegal	Two
33	Equatorial Guinea	None
34	Liberia	None
35	Serra Leone	None
36	Madagascar	None
37	Seychelles	None
38	Guinea	None
39	Guinea Bissau	None
40	Saharawi	None
41	Cabo Verde	None
42	Burundi	None
43	Burkina Fasso	Three
44	Zimbabwe	Three
45	Eswatini	None
46	Comoros	None
47	Somalia	None
48	Djibouti	None
49	São Tomé and Príncipe	None
50	Mauritius	None
51	Gabon	None
52	Central African Republic	None
53	Mauritania	None
54	Benin	None

Title: **Peripartum cardiomyopathy: A review of prevalence and treatment trends from an African perspective**

Table 2: summary of the number of articles retrieved from the 54-African Countries

Search platform: **Scopus**

Search keywords: Peripartum cardiomyopathy (PPCM)+ (per Lists of African countries),

Article searched (Duration of publication): till November 17th, 2024

Manuscript title	Focus of the article	Number of patients with PPCM
Medium term prognosis of Egyptian patients hospitalised with acute decompansated heart failure	Studied parameters that affect heart failure prognosis in hospitalized patients	Six patients out of 100 study participants
Characterization of Non-Ischemic Dilated Cardiomyopathy in a Native Tanzanian Cohort: MOYO Study	characterized NIDCM in a Tanzania with respect to demographics, clinical profile, imaging findings and management	Forty-five patients (11.2%) were those with peripartum cardiomyopathy
Burden, predictors and short-term outcomes of peripartum cardiomyopathy in a black African cohort	investigate the prevalence, predictors and six-month outcomes of PPCM patients	It found the prevalence of PPCM to be 17.4 %, (41 patients out of 236 patients participated in the study).
Left ventricular recovery in an African cohort of patients with peripartum cardiomyopathy	left ventricular recovery and poor outcome at one year of PPCM	All the study participants were those with PPCM (94 patients studied)
Peripartum cardiomyopathy in patients with psychiatric disorders successfully treated with bromocriptine: Two case reports	cases of two patients with PPCM and mental disorders treated with bromocriptine therapy. The first was 33-year-old with a history of atypical depression and anxiety disorder and PPCM, (LVEF=19%), the next was 42-year-old with a history of bipolar and panic disorders with PPCM (LVEF=18%).	Both treated with bromocriptine; psychiatric symptoms did not worsen and cardiac function improved
Five cases of Peripartum Cardiomyopathy in Malawi	five cases of peripartum cardiomyopathy, presented to the hospital over an 18-months period.	The patients were followed and their treatment outcomes were documented.

Serum selenium and dilated cardiomyopathy in Cotonou, Benin	This study measured serum selenium levels in patients PPCM and DCM and compare patients with healthy postpartum women with comparable or identical obstetric features.	It found that Selenium was similar in both ppcm and DCM patients, generally lower than their normal counterparts. The study included ten patients with PPCM
Epidemiological features and mortality risk factors of peripartum cardiomyopathy in a group of Sub-Saharan African population	The hospital frequency of PPCM was 1.3%. Dyspnea was present in all patients, 89.7% of them had LEDD of ≥ 62 m, 48.3% had LVEF between 30% and 45%, while 51.7% had an LVEF < 30%.	Majority of patients with PPCM were from rural area, with Mortality rate 27.7%, with age less than 30 years was independently associated with mortality.
Peripartum cardiomyopathy: Characteristics and outcomes among women seen at a referral hospital in Lusaka, Zambia	The study described the phenotype of women with PPCM and examined outcomes at 6 months following recruitment.	The study found that out of 45 participants with PPCM, 38 were seen at 6-month follow up of whom 7 % died.
Prevalence and characteristics of dysfunction of right ventricle in peripartum cardiomyopathy	The study assessed the prevalence of RV dysfunction in a PPCM population.	The study found that 19 out of 326 patients hospitalized were ppcm, making a hospital prevalence of 5.8%. Furthermore, it found that RV dysfunction was common in PPCM
Peri-Partum Cardiomyopathy: Epidemiological, Clinical Aspects and Risk Factors in Semi-Urban Areas in Senegal	The study found that there were 32 cases of PPCM with average age average age was 27.19 ± 8.082 years, making inpatient prevalence of 2.3%.	The study had 32 patients with ppcm of the population studied.
Peripartum cardiomyopathy among cardiovascular patients referred for echocardiography at Parirenyatwa Teaching Hospital, Harare, Zimbabwe	It evaluated the outcome of patients with PPCM within six months of diagnosis and describe demographic and clinical characteristics.	It found the death at six-month being 11.6%, which showed 5 out of 43 participants died.
Epidemiology of Peripartum Cardiomyopathy in Africa	The study reviewed the epidemiology of the disease in Africa with limited details.	The multicenter study conducted in Kano, Nigeria, included 1296

		patients who were referred for echocardiography over a period of 7 months. 55 individuals were diagnosed with peripartum cardiomyopathy. This indicates that PPCM was the most prevalent type of cardiomyopathy found in this specific study, accounting for 52.4% of all cardiomyopathies diagnosed during that time.
Clinical characteristics and long-term outcome of peripartum cardiomyopathy in a resource limited setting.	The study evaluated clinical outcomes and risk factors for adverse outcomes among PPCM patients.	It showed that 49% of 49 of patients had full LVEF recovery in the study duration.
Clinical presentation, management, and 6-month outcomes in women with peripartum cardiomyopathy: An ESC EORP registry	It described the clinical presentation, management, and 6-month outcomes in women with peripartum cardiomyopathy (PPCM) globally, in which Africans were also included.	It included 29% of Seven hundred and thirty-nine women from Africa, mean age was 31 ± 6 years, mean LVEF was 31 ± 10%.
Impact of pregnancy-related heart failure on humoral immunity: Clinical relevance of G3-subclass immunoglobulins in peripartum cardiomyopathy	It studied the relative impact of pregnancy-related onset of HF on humoral immunity.	It found that immunoglobulins, frequencies and reactivities, were markedly raised in PPCM patients compared with DCM.
Maternal and fetal prognosis of subsequent pregnancy in black African women with peripartum cardiomyopathy	It described maternal and fetal outcomes after pregnancy complicated by peripartum cardiomyopathy (PPCM), where 29 ppcm, with a mean age of 26.7 ± 4.6 years, mean gravidity of 2.3 ± 0.5 and the mean LVDD was 53 ± 4 mm and LVEF was ≥50% in 13 cases (44.8%).	It showed 14 deaths (48.3%), SSP outcomes are still severe with maternal mortality remains high.

Bromocriptine in Management of Peripartum Cardiomyopathy: A Randomized Study on 96 Women in Burkina Faso	This study assessed the role of bromocriptine in management of peripartum cardiomyopathy in Burkina Faso.	It randomized 96 ppcm with bromocriptine treatment.
Prognosis of peripartum cardiomyopathy in sub-Saharan Africa (Burkina Faso South-West PPCM register)	The study followed 60 patients with PPCM for 1 year, determined the factors associated with the non-recovery of myocardial function upon 12 months of diagnosis.	It found mortality rate of 13.3%, with delay diagnosis and observance being related with non- recovery
Outcome in German and South African peripartum cardiomyopathy cohorts' associates with medical therapy and fibrosis markers	This study compared the clinical course of peripartum cardiomyopathy cohorts from Germany (G-PPCM) and South Africa (SA-PPCM) with fibrosis-related markers.	SA-PPCM displayed a more profibrotic biomarker, which was associated with a less favorable outcome
Outcome of subsequent pregnancies in patients with a history of peripartum cardiomyopathy	It studied the outcome of SSPs in PPCM patients in Germany, Scotland, and South Africa.	Showed increased risk of ppcm on subsequent pregnancy with worse outcome.
Poor outcome of indigent patients with peripartum cardiomyopathy in the United States	This study analyzed the clinical and echocardiographic data of 44 (39 African American) patients with PPCM over an 11-year period (1992-2003).	Limited data.
The importance of cardiovascular pathology contributing to maternal death: Confidential enquiry into maternal deaths in South Africa, 2011-2013	It determined the cardiovascular causes and contributing factors of maternal death.	It found PPCM is one of the cardiovascular causes of maternal death.
Impact of pregnancy-related heart failure on humoral immunity: Clinical relevance of G3-subclass immunoglobulins in peripartum cardiomyopathy	It studied the relative impact of pregnancy-related onset of HF on humoral immunity.	It found Immunoglobulins against cardiac myosin in 47 patients with PPCM.
The 12-lead ECG in peripartum cardiomyopathy	To determine the prevalence of major and minor ECG abnormalities in PPCM patients at the time of diagnosis, and to	ECG patterns among 78 ppcm

	establish whether there are ECG correlates of persistent left ventricular dysfunction and/or clinical stability at six months of follow up, where available	
Discoveries in peripartum cardiomyopathy	emphasizes the importance of continuing the process of increasing awareness of PPCM and presents details of this evolving picture, including important discoveries that point the way to full recovery for almost all PPCM subjects	Thus, there were 48 participants in the study, but none of them had peripartum dilated cardiomyopathy.
Sunday, 29 August 2010	It defined the risk factors for development of PPCM and the predictors of survival outcome in PPCM.	It studied 30 patients with PPCM.
Low systolic blood pressure and high resting heart rate as predictors of outcome in patients with peripartum cardiomyopathy	It studied prediction of risk and the contribution of high resting heart rate (HR) and low SBP to risk in recent onset of PPCM.	It showed high resting heart rate (HR) and low SBP might be associated with risk in development of PPCM.
Predictors of outcome in 176 South African patients with peripartum cardiomyopathy	It tried to identify novel prognostic factors for patients with peripartum cardiomyopathy (PPCM)	176 African women with newly diagnosed PPCM were studied.
Interventions for treating peripartum cardiomyopathy to improve outcomes for women and babies	It assessed the effectiveness and safety of any intervention for the care of women and/or their babies with a diagnosis of peripartum cardiomyopathy.	The study does not specify the exact number of patients included in the study or how many of them had peripartum dilated cardiomyopathy. However, it predicted the incidence of PPDCM being 1000 to 1300 cases each year.
ECG characteristics in peripartum cardiomyopathy	It assessed the prevalence of ECG abnormalities in newly diagnosed PPCM patients at baseline and at 6 months of follow-up	The study included 39 consecutive patients diagnosed with peripartum cardiomyopathy (PPCM) who presented to a tertiary center in South Africa. It

		was noted that 93% of these patients had abnormal ECGs, which indicates a high prevalence of cardiac abnormalities among those diagnosed with PPCM.
Risk factors for disease development and predictors of outcome in peripartum cardiomyopathy	It defined the risk factors for development of PPCM and the predictors of survival outcome in PPCM	The paper discusses peripartum cardiomyopathy (PPCM) as a serious condition affecting young women, but it does not specify the exact number of patients who had peripartum dilated cardiomyopathy within the study group. However, it highlights the incidence can be as high as 1 in 100 births in Nigeria.
Familial aggregation of dilated cardiomyopathy in patients with peripartum cardiomyopathy	report on a study of the familial aggregation of DCM in patients with PPCM	It studied the possible familial nature of PPCM.
The importance of cardiovascular pathology contributing to maternal death: Confidential enquiry into maternal deaths in South Africa, 2011-2013	determine the cardiovascular causes and contributing factors of maternal death in South Africa, and identify avoidable factors, and thus improve the quality of care provided	In the study, a total of 169 maternal deaths were reported for the period from 2011 to 2013 in South Africa. Out of these, 118 complete hospital case files were available for analysis. Among these analyzed cases, 34% (40) were attributed to peripartum cardiomyopathy (PPCM).
Peripartum cardiomyopathy: A review of the literature	Reviewed ppcm	Full data Can't be accessible
Prevalence of peripartum	Review of ppcm in pregnancy.	The case report you provided

cardiomyopathy in pregnant women		describes a single patient who was diagnosed with peripartum cardiomyopathy (PPCM) after presenting with shortness of breath and developing asthma cardinals shortly after spontaneous delivery.
Peripartum cardiomyopathy: Current state of knowledge, new developments and future directions	The present article revisits these concepts and recent advances in PPCM.	It highlights that PPCM is a distinct form of idiopathic dilated cardiomyopathy affecting women during late pregnancy or shortly after childbirth. It notes the variability in incidence and prognosis based on geography and emphasizes that many aspects of the disease, including its incidence, etiology, and optimal treatment, remain poorly understood.
Incidence, clinical characteristics, and risk factors of peripartum cardiomyopathy in Nigeria: results from the PEACE Registry	It described the incidence, clinical characteristics and risk factors of peripartum cardiomyopathy (PPCM) in Nigeria.	The study you referenced involved 406 patients who were diagnosed with peripartum cardiomyopathy (PPCM) and 99 controls who were healthy women who had recently delivered.<br><br>As the focus of the study was on describing the incidence, clinical characteristics, and risk factors of PPCM in Nigeria, all 406 patients had peripartum dilated cardiomyopathy (PPCM).
Learning from the peripartum	It determined the burden and	In the PEACE registry study, a

cardiomyopathy in Nigeria (PEACE) registry: A multisite, contemporary PPCM registry in Nigeria	demographic, clinical characteristics, myocardial remodeling, and survival of peripartum cardiomyopathy (PPCM) in Nigeria.	total of 1,000 patients were included, among which 65% (or approximately 650 patients) were diagnosed with peripartum dilated cardiomyopathy (PPCM).
Serum selenium and ceruloplasmin in Nigerians with peripartum cardiomyopathy	It determined if selenium deficiency, serum ceruloplasmin and traditional birth practices are risk factors for peripartum cardiomyopathy (PPCM)	The study you referenced involved 39 PPCM patients and 50 controls. All 39 patients in the study had peripartum dilated cardiomyopathy (PPCM), as the study aimed to investigate risk factors for PPCM in Kano, Nigeria. The patients were followed up for six months to assess various factors associated with the disease, such as selenium deficiency and serum ceruloplasmin levels.
Rationale and design for the peripartum cardiomyopathy in Nigeria (PEACE) registry	It described the burden, ventricular remodeling, and outcomes (rehospitalization rate, cardioembolic events and survival) of PPCM in Nigeria.	The main study and its sub-studies involve large cohorts, including 200 PPCM patients for selenium treatment and 120 healthy pregnant women.<br>For the PPCM patients, the exact number in the main registry isn't stated, but it's clear that they had peripartum dilated cardiomyopathy (PPCM) as part of the study's focus.
Clinical Features and Outcomes of Peripartum Cardiomyopathy in Nigeria	It examined the clinical profile, myocardial remodeling, and survival of patients with PPCM in Nigeria.	In the study "Clinical Features and Outcomes of Peripartum Cardiomyopathy in Nigeria," a total of 1,000 patients were included. Out of these, 650

		patients (65%) were diagnosed with peripartum dilated cardiomyopathy (PPCM).
Disparities in clinical features and outcomes of peripartum cardiomyopathy in high versus low prevalent regions in Nigeria	It determined if this phenomenon was characterized by a differential case profile and outcome among PPCM cases originating elsewhere	The study recruited PPCM patients from 14 study centers in Nigeria between June 12, 2017, and March 31, 2018. However, the exact number of patients included in the study and the specific count of those diagnosed with peripartum dilated cardiomyopathy (PPCM) is not explicitly stated in the provided context.
Worldwide incidence of peripartum cardiomyopathy and overall maternal mortality.	Reviewed worldwide prevalence of ppcm	The study on peripartum cardiomyopathy (PPCM) reviewed various reports to summarize the incidence of this condition across different countries. The study highlighted that the incidence of PPCM is particularly high among African-Americans in the United States, with rates ranging from 1 in 439 to 1,421 deliveries. This indicates a relative risk approximately 16 times higher than that of other races
Right ventricular systolic dysfunction and remodeling in Nigerians with peripartum cardiomyopathy: A longitudinal study	assess RVSD and remodeling in a cohort of PPCM patients in Kano, Nigeria	The study involved 45 patients who were recruited over 6 months. These patients had peripartum cardiomyopathy (PPCM), and the study aimed to assess right ventricular systolic

		dysfunction (RVSD) and remodelling in this cohort. The study shows that RVSD was common in these PPCM patients, and a significant portion experienced recovery over the 12-month period.
Peripartum cardiomyopathy	Review on ppcm	Limited data
One-year survival in Nigerians with peripartum cardiomyopathy. 20	describe the 1-year survival and left ventricular reverse remodeling (LVRR) in a group of patients with PPCM from three referral hospitals in Kano, Nigeria.	The study involved 45 patients who were recruited over 6 months. These patients had peripartum cardiomyopathy (PPCM), and the study aimed to assess right ventricular systolic dysfunction (RVSD) and remodelling in this cohort.
Regional disparities in the clinical profiles of patients with peripartum cardiomyopathy in Nigeria: results from the peripartum cardiomyopathy in Nigeria Registry	describe the regional differences (if any) in the clinical profiles of patients with PPCM in Nigeria	The study included a total of 244 PPCM patients, with 199 patients (81.6%) recruited from 3 hospitals in Kano, and 45 patients (18.4%) from 11 hospitals across other regions in Nigeria (North-Central, South-West, South-East, and South-South). The study aimed to describe the regional differences in the clinical profiles of PPCM patients in Nigeria. 35 patients (17.6%) from Kano and 10 patients (23.2%) from other regions died after a median follow-up of 17 months.
Clinical profile and outcomes for	study the clinical profile, myocardial	The paper does not provide

peripartum cardiomyopathy in Nigeria: a prospective longitudinal study	remodeling and survival of patients with PPCM in Nigeria	specific information regarding the prevalence or number of patients with peripartum dilated cardiomyopathy.
Prevalence and characteristics of apparent resistant hypertension in patients at Kenyatta national hospital medical outpatient clinic	explored the relationship between serum selenium and ventricular dysfunction in apparently healthy pregnant women, and the impact of selenium supplementation on left ventricular reverse remodeling (LVRR), change in symptoms and survival in an open-label randomized trial.	The study included a total of 244 patients diagnosed with peripartum cardiomyopathy (PPCM) in Nigeria highlighting the high incidence of PPCM in Nigeria, which is noted to be the highest in the world.
Prevalence and Characteristics of Peripartum Cardiomyopathy among Women with Cardiac Failure Referred for Echocardiography in a Tertiary Hospital in Northern Nigeria	determine the prevalence and characteristics of PPCM among women with heart failure referred for echocardiography	The study included a total of 401 women who were referred for echocardiography due to heart failure between October 2016 and September 2017. Among these, 256 women (69.5%) were diagnosed with peripartum cardiomyopathy (PPCM), making it the most common cause of heart failure in the study population.
Electrocardiographic predictors of peripartum cardiomyopathy	identify potential electrocardiographic predictors of peripartum cardiomyopathy (PPCM)	The researchers recruited a total of 131 patients. This included 54 patients diagnosed with peripartum cardiomyopathy (PPCM) and 77 control patients , providing valuable insights into the electrocardiographic predictors associated with this condition.
Epidemiologic profile of peripartum	describe the current epidemiologic profile	The study involved 65 patients

cardiomyopathy in a tertiary care hospital	of PPCM	who were all diagnosed with peripartum cardiomyopathy (PPCM)
ABO-Rhesus Blood Group Distribution among Peri-Partum Cardiomyopathy Patients: A Multi-Center Study in Sokoto, Nigeria 27	study assessed the distribution of the different ABO-Rh groups among ninety patients diagnosed with PPCM	The study included a total of 90 patients who were diagnosed with peripartum cardiomyopathy (PPCM) from three centers in Sokoto metropolis. All 90 participants were diagnosed with PPCM.
Peripartum cardiomyopathy in Nigeria: A historical perspective	summarize the historical aspect of PPCM report and care in Nigeria	The article does not provide specific numerical data regarding the total number of patients involved in the study or the exact number of patients diagnosed with peripartum dilated cardiomyopathy. However, it does mention that Ian Fraser Brockington was the first to publish on PPCM in Nigeria, reporting on the clinical profile of 50 cases of postpartum heart failure that presented at the University College Hospital Ibadan during the years 1962, 1965, and 1967-1969.
Selenium supplementation in patients with peripartum cardiomyopathy: a proof-of-concept trial	assessed the effects of selenium supplementation in PPCM patients who had selenium deficiency in Nigeria	The study included 100 patients with peripartum cardiomyopathy (PPCM), all of whom had a left ventricular ejection fraction (LVEF) of <45% and selenium deficiency (<70 μg/L).
Peripartum cardiomyopathy: A review	discusses the epidemiology, risk factors,	Mentioned that PPCM was

article 30	aetiology, clinical features, diagnosis, treatment and prognosis of PPCM	found in 55 out of 1296 patients referred for echocardiography in Kano, Nigeria, representing 4.2% of all cardiomyopathies diagnosed during that period.<br>This indicates that a substantial number of patients were analyzed, and a notable proportion had PPCM.
Right ventricular systolic function in peripartum and dilated cardiomyopathies	assess and compare RV systolic function between PPCM and idiopathic DCM, using tricuspid annular plane systolic excursion (TAPSE)	Limited data.
Peripartum cardiomyopathy	Overview of ppcm	
Profile of peripartal cardiomyopathy seen in an academic hospital in south west Nigeria	Local registry in January 2012 at our center to properly profile the cases of PPCM	The study analyzed 65 patients diagnosed with peripartum cardiomyopathy (PPCM).<br>Among these, 27 patients (41.5%) had severe left ventricular dysfunction, which is characteristic of peripartum dilated cardiomyopathy (defined by left ventricular end-diastolic dimension >6 cm and fractional shortening <20%).
Abstract 8991: Prognostic Significance of Hyponatremia in Peripartum Cardiomyopathy: Insights from the Peripartum Cardiomyopathy in Nigeria Registry	hyponatremia will have prognostic significance in PPCM patients	The study involved 191 patients in total. Among these, all patients had peripartum cardiomyopathy (PPCM) because the study specifically focused on patients diagnosed with PPCM. The distinction within the study lies in

		comparing PPCM patients with hyponatremia (90 patients) and those with normal serum sodium levels (101 patients).
Influence of systolic blood pressure on outcomes in Nigerians with peripartum cardiomyopathy	assess the clinical features and outcomes (all-cause mortality and unrecovered left ventricular [LV] systolic function) of PPCM patients grouped according to their baseline systolic BP (SBP)	The study recruited 227 patients, all of whom had peripartum cardiomyopathy (PPCM). The study found that 20% of PPCM patients were hypotensive at presentation, with a systolic blood pressure (SBP) of less than 90 mmHg, which was associated with a significantly higher risk of all-cause mortality (4-fold higher). However, SBP at presentation was not associated with recovery of left ventricular (LV) systolic function. This suggests that low BP at presentation in PPCM patients may indicate a poorer prognosis, particularly in terms of mortality, but not necessarily affect LV function recovery.
Prevalence and predictors of right ventricular diastolic dysfunction in peripartum cardiomyopathy	assess the prevalence of right ventricular diastolic dysfunction (RVDD) and its potential predictors in peripartum cardiomyopathy (PPCM) patients	The study involved 43 patients, all of whom had peripartum cardiomyopathy (PPCM). The study found that right ventricular diastolic dysfunction (RVDD) was present in 69.8% of PPCM patients, with a significant portion of these patients having grade I (53.3%) or grade II (40.0%) severity. Additionally,

		right ventricular systolic dysfunction (RVSD) was observed in 88.4% of patients, and combined RVSD and RVDD was found in 58.1%. Lower serum selenium levels (<70 µg/L) and the presence of RVSD with pulmonary hypertension were identified as significant predictors of RVDD.
Baseline clinical profile of chronic heart failure in Nigeria: result of a national registry	describe the clinical profile and characteristics of chronic HF patients attending the out-patients' services of selected hospitals in Nigeria	The study involved 452 consecutive patients presenting with acute heart failure (AHF), but none of them were reported to have peripartum dilated cardiomyopathy.
Heart Failure Admissions in Medical Wards ofa Nigerian Tertiary Hospital	determined the prevalence, pattern and mortality rate of heart failure among patients admitted in to medical wards of Murtala Muhammad Specialist Hospital (MMSH) Kano, a tertiary hospital in North Western Nigeria.	The study involved 1,651 patients, out of which 268 (16.2%) were admitted due to heart failure. Of these, 38.9% had peripartum cardiomyopathy (PPCM), and 6.3% had dilated cardiomyopathy (DCM).<br>The study highlights the high prevalence of heart failure in Kano, Nigeria, with hypertensive heart disease, peripartum cardiomyopathy, and dilated cardiomyopathy as the main etiologies, emphasizing the need for better hypertension management and further research on PPCM.

Pattern and clinical characteristics of cardiomyopathies among adults in Kano, Nigeria: An interim report	determine the pattern and clinical characteristics of cardiomyopathies in Kano, Nigeria.	Limited data. An interim report.
Prevalence of Left Ventricular Dysfunction and Relationship with Serum Selenium in Apparently Healthy Pregnant Women: Results from the PEACE Registry	determine the prevalence of left ventricular (LV) systolic and diastolic dysfunction in apparently healthy pregnant women and if there is relationship with serum selenium, in a society with high prevalence of peripartum cardiomyopathy (PPCM)	Total participants: 108. Those with LV systolic dysfunction (a potential indicator of PPCM): 6 during pregnancy (5.6%) and 9 after delivery (10.2%).
Factors Associated with Intra-hospital Mortality of Peripartum Cardiomyopathy Patients in Northcentral Nigeria	studied the patient characteristics, intra-hospital outcomes and factors associated with intra-hospital mortality in patients admitted for Peripartum Cardiomyopathy (PPCM) in our center using data from the Ilorin Heart failure Registry.	The study included 22 confirmed peripartum cardiomyopathy (PPCM) patients. In PPCM patients, intra-hospital mortality was associated with lower serum sodium, eGFR, ejection fraction (EF), and fractional shortening (FS), highlighting the need for targeted interventions in high-risk subgroups.
Prevalence of peripartum cardiomyopathy in pregnant women.	The reported incidence of peripartum cardiomyopathy varies due to wide geographical variation, with reported incidences of 1:2289 to 1:4000 live births in the United States, 1:1000 in South Africa, 1:300 in Haiti, and 1:100 in Zaria and Nigeria.	The study included 8,100 pregnancies, and the incidence of peripartum cardiomyopathy was found to be 1:2,700 live births.<br>Cardiovascular disease during pregnancy and the postpartum period is significant and requires careful management, as evidenced by the incidence of peripartum cardiomyopathy.

Title: **Peripartum cardiomyopathy: A review of prevalence and treatment trends from an African perspective**

Table 3: search summary of the number of articles retrieved from African studies

Search platform (Database): Ovid

Search keywords: Peripartum cardiomyopathy (PPCM)+ (per Lists of African countries),

Article searched (Duration of publication): till November 17th, 2024

Database: summary (Ovid MEDLINE(R) Epub Ahead of Print <January 15, 2025)

#	Query	Results from 16 Jan 2025
1	..nlp peripartum cardiomyopathy in Africa {Including Related Terms}	3,037
2	..nlp PPCM Africa {Including Related Terms}	3,029
3	limit 11 to (English language and full text and "remove preprint records")	146
4	limit 12 to yr="2004 - 2024"	126

https://ovidsp.ovid.com/ovidweb.cgi?T=JS&NEWS=N&PAGE=main&SHAREDSEARCHID=rbUNRX2HMTwCregV44JErBuOAKWA9rMeDlv6gPUmnnudYzrA9CYe4APf1WIg0poO

Title: Peripartum cardiomyopathy: A review of prevalence and treatment trends from an African perspective

Table 4: Prevalence of peripartum cardiomyopathy in African studies

**Title of the Article**	**Reported prevalence of peripartum cardiomyopathy**
Characterization of Non-Ischemic Dilated Cardiomyopathy in a Native Tanzanian Cohort: MOYO Study	11.2%
Burden, predictors and short-term outcomes of peripartum cardiomyopathy in a black African cohort	17.4
Epidemiological features and mortality risk factors of	1.3 (It was hospital prevalence)

peripartum cardiomyopathy in a group of Sub-Saharan African population	
Prevalence and characteristics of dysfunction of right ventricle in peripartum cardiomyopathy	5.8 (It was hospital prevalence)
Peri-Partum Cardiomyopathy: Epidemiological, Clinical Aspects and Risk Factors in Semi-Urban Areas in Senegal	2.3 (It was hospital prevalence)
Prevalence of peripartum cardiomyopathy in pregnant women	1:2289 to 1:4000 live births (USA)
	1:1000 (South Africa)
	1:300 (Haiti)
	1:100 (Zaria)
	1:100 (Nigeria)

Title: Peripartum cardiomyopathy: A review of prevalence and treatment trends from an African perspective

Table 5: summary of the number of articles retrieved from African Countries showing mortality rates among patients with PPCM

Article searched (Duration of publication): till November 17th, 2024

Manuscript title	Mortality rate reported	Bromocriptine use
Peripartum cardiomyopathy in patients with psychiatric disorders successfully treated with bromocriptine: Two case reports	cases of two patients with PPCM treated with bromocriptine therapy, (LVEF=19% and 18%).	Cardiac function improved in both of them.
Five cases of Peripartum Cardiomyopathy in Malawi	five cases of peripartum cardiomyopathy: the details in the body of this manuscript.	The bromocriptine was used in two of them.
Epidemiological features and mortality risk factors of peripartum cardiomyopathy in a group of Sub-Saharan African population	Mortality rate 27.7%, with age less than 30 years was associated with mortality.	Not documented.
Peripartum cardiomyopathy: Characteristics and outcomes among women seen at a referral hospital in Lusaka, Zambia	7% of them died.	Noted documented.
Peripartum cardiomyopathy among cardiovascular patients referred for echocardiography at Parirenyatwa	It found the death at six-month being 11.6%.	Not reported.

Teaching Hospital, Harare, Zimbabwe		
Maternal and fetal prognosis of subsequent pregnancy in black African women with peripartum cardiomyopathy	It showed 48.3%, SSP outcomes are still severe with maternal mortality remains high.	Not reported.
Bromocriptine in Management of Peripartum Cardiomyopathy: A Randomized Study on 96 Women in Burkina Faso	This study assessed the role of bromocriptine in management of peripartum cardiomyopathy.	It randomized 96 ppcm with bromocriptine treatment.
Prognosis of peripartum cardiomyopathy in sub-Saharan Africa (Burkina Faso South-West PPCM register)	It found mortality rate of 13.3%, with delay diagnosis being related with non-recovery	Not reported.
